# Bioinformatics analysis reveals TSPAN1 as a candidate biomarker of progression and prognosis in pancreatic cancer

**DOI:** 10.17305/bjbms.2020.5096

**Published:** 2021-02

**Authors:** Chenhui Ma, ZeLong Cui, YiChao Wang, Lei Zhang, JunYe Wen, HuaiBin Guo, Na Li, WanXing Zhang

**Affiliations:** 1 Department of Hepatobiliary, Hebei General Hospital, Shijiazhuang, China; 2 Graduate School of North China University of Science and Technology, Tangshan, China; 3 Department of Hematology, Qilu Hospital of Shandong University, Jinan, China

**Keywords:** Pancreatic cancer, TSPAN1, TCGA, GEO dataset, diagnosis and prognosis

## Abstract

Pancreatic cancer (PCC) is a common malignant tumor of the digestive system that is resistant to traditional treatments and has an overall 5-year survival rate of <7%. Transcriptomics research provides reliable biomarkers for diagnosis, prognosis, and clinical precision treatment, as well as the identification of molecular targets for the development of drugs to improve patient survival. We sought to identify new biomarkers for PCC by combining transcriptomics and clinical data with current knowledge regarding molecular mechanisms. Consequently, we employed weighted gene co-expression network analysis and differentially expressed gene analysis to evaluate genes co-expressed in tumor versus normal tissues using pancreatic adenocarcinoma data from The Cancer Genome Atlas and dataset GSE16515 from the Gene Expression Omnibus. Twenty-one overlapping genes were identified, with enrichment of key Gene Ontology and Kyoto Encyclopedia of Genes and Genomes pathways, including epidermal growth factor receptor signaling, cadherin, cell adhesion, ubiquinone, and glycosphingolipid biosynthesis pathways, and retinol metabolism. Protein-protein interaction analysis highlighted 10 hub genes, according to Maximal Clique Centrality. Univariate and multivariate COX analyses indicated that TSPAN1 serves as an independent prognostic factor for PCC patients. Survival analysis distinguished TSPAN1 as an independent prognostic factor among hub genes in PCC. Finally, immunohistochemical staining results suggested that the TSPAN1 protein levels in the Human Protein Atlas were significantly higher in tumor tissue than in normal tissue. Therefore, TSPAN1 may be involved in PCC development and act as a critical biomarker for diagnosing and predicting PCC patient survival.

## INTRODUCTION

Pancreatic cancer (PCC) is a highly immunosuppressive and malignant tumor of the digestive system, with insidious onset, rapid disease progression, and no breakthroughs in long-term efficacy or prognosis. In a recent report, PCC ranked 10^th^ among cancers in the United States for its incidence but 4^th^ for its mortality [1]. Furthermore, it is estimated to reach the second largest cause of cancer-related death by 2030 [2]. In China, PCC does not rank among the top 5 in mortality. However, the proportion of death caused by PCC has increased by 9% over the past 10 years, primarily accounted for by changes in lifestyle and diet and the acceleration of population aging [3]. The specific pathogenesis of PCC remains unclear, but a large number of clinical and epidemiological findings have revealed that smoking and obesity prolong pancreatitis, and diabetes has been identified as a significant independent risk factor for the development of PCC [4]. PCC progresses rapidly, and early detection and diagnosis are crucial for the prognosis of PCC patients [4]. Nonetheless, imaging examination, serological markers, and other diagnostic methods have limitations, especially for early PCC diagnosis, which compromises clinical care and prognosis [4,5]. The major strategies of PCC management include surgery, chemotherapy, radiotherapy, molecular guided therapy, and immunotherapy [4]. However, due to PCC’s pathological and clinical characteristics, chemotherapy and radiotherapy have little benefit for patients with PCC, and currently, the most successful therapeutic choice for PCC is surgical resection [6]. Through the rapid growth and comprehensive implementation of gene detection technology, molecularly targeted drugs have been increasingly used in clinical practice. Nevertheless, molecular targeted therapy has not been successfully applied to PCC due to the poor understanding of its molecular pathological mechanism [7].

For a thorough and rigorous understanding of PCC, a clinical risk assessment needs to be combined with clinical characteristics. For example, the cancer stage, diagnostic grade, and cancer laterality in PCC are correlated with the patient’s diagnostic age, overall survival (OS), and secondary malignancies [8]. The rapid development of microarray and sequencing technology provides a useful method and forum for the research of cancer and other diseases [9]. New biomarkers for diagnosis, treatment, and prognosis can be obtained by combining clinical data with molecular mechanisms [10]. Weighted gene co-expression network analysis (WGCNA) provides an approach for performing weighted network analysis in the R package. It can be used in research to characterize multiple sample and cluster-specific gene expression patterns and to detect highly related gene expression modules correlated with clinical characteristics [11]. WGCNA is also useful for identifying core genes as well as the function of co-expressed genes of tumors and other diseases [12,13]. For instance, Liu et al. successfully identified five lncRNAs associated with survival in hepatocellular carcinoma by co-expression analysis [14]. Differential gene expression analysis based on transcriptomics offers substantial insight into the molecular mechanisms of genome-regulated diseases, the transcriptional behavior of biological systems, and potential biomarkers for specific diseases [15].

We used WGCNA to explore and evaluate the etiology and molecular characteristics of PCC in a systematic manner, to measure transcriptional expression levels, and identified differentially expressed genes (DEGs) in PCC from The Cancer Genome Atlas (TCGA) and Gene Expression Omnibus (GEO) databases. Moreover, we combined the DEG results with functional enhancement and protein-protein interaction (PPI) analysis, survival analysis, and Cox regression, and identified DEGs closely related to the prognosis of PCC. These studies constitute a basis for drug development and a potential reference for the clinical diagnosis and treatment of PCC.

## MATERIALS AND METHODS

### Study design

**FIGURE 1:**
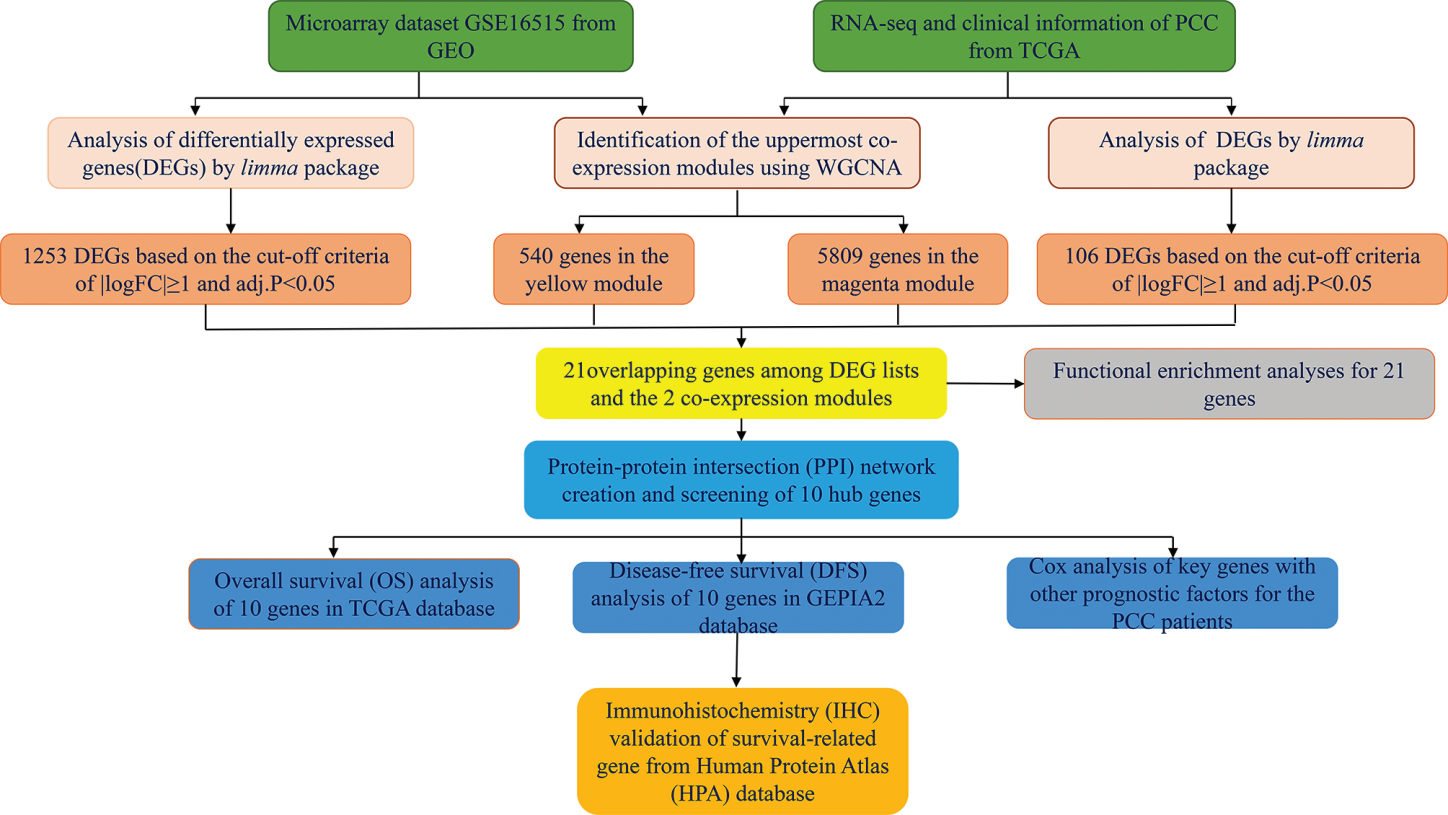
Overview of the study design. DEGs and co-expression modules in PCC were identified from microarray data from GEO (GSE16515); and RNA-seq and clinical data from TCGA-PAAD. Twenty-one overlapping genes were further evaluated by functional enrichment and protein-protein intersection analysis, and 10 Hub genes were identified. The correlations of these Hub genes with OS, DFS, and prognostic factors were assessed. IHC data from HPA were evaluated for further validation.

A detailed workflow of our study design is shown in Figure 1. We analyzed microarray data from GEO (GSE16515) and RNA-seq and clinical data from TCGA. The sets of DEGs identified by the limma R package, and the most highly co-expressed modules, which were identified by WGCNA, were evaluated for overlap. The 21 overlapping genes were subjected to functional analysis and PPI analysis. The correlation of the overlapping genes and clinical parameters, including OS, disease-free survival (DFS), and other prognostic factors, was evaluated, and immunohistochemistry (IHC) data were assessed to validate the expression of survival-related genes in the Human Protein Atlas (HPA).

### Data collection and preprocessing

The GSE16515 dataset was accessed from the GEO database (http://www.ncbi.nlm.nih.gov/geo/). We selected this dataset because it included stringent screening criteria and has gene expression profiles of 52 PCC samples that were obtained using the GPL570-55999 ([HG-U133_Plus_2] Affymetrix Human Genome U133 plus 2.0 Array), which is a well-established platform [16]. According to the manufacturer’s Annotation document, the probes were first assigned corresponding genetic symbols, and then the median of all associated findings was calculated to exclude detection overlap for the same gene. Consequently, 21654 genes were evaluated. An additional set of RNA-sequencing data of 182 PCC samples was downloaded from the TCGA database (https://genome-cancer.ucsc.edu/) from the pancreatic adenocarcinoma (PAAD) cohort, along with full expression profiles and data on clinically relevant characteristics. The TCGA data were annotated using a Human hg38 gene track reference transcript array. As indicated by the edgeR package tutorial [17], genes with low read counts were not useful for further study. Therefore, in this study, the genes with CPM (count per million) at or above 1 were analyzed. In subsequent analysis, a total of 15035 genes with RPKM values were analyzed, and the RPKM function was filtered by the edgeR software package, which distinguishes the number of genes according to the gene length.

### DEG analysis

The limma R package [18] provides an efficient approach for differential expression analysis of microarray and RNA-sequencing data. Therefore, it was utilized in this study to screen DEGs between non-malignant pancreatic samples and PCC tissues. DEGs were defined as genes with the cutoff criteria of |logFC| ≥ 1.0 and adj. *p* < 0.05. In the ggplot2 package in R, the DEGs of the TCGA-PAAD and GSE16515 datasets were presented through volcano plots [19].

### Construction and identification of a gene co-expression network by WGCNA

Quality assessment of the data was performed, and a gene co-expression network was established using the WGCNA package in R for DEGs [20]. WGCNA reveals heavily clustered gene modules between specimens and connects the modules to outer template characteristics. Before building the network, the number of genes with different thresholds of expression was estimated, and the pickSoftThreshold function was used to construct a scale-free network. Next, the formula aij = |Sij|β (aij: matrix of adjacency between gene i and gene j, Sij: Matrix of similarity made by Pearson correlation of all gene pairs) was used to construct an adjacency matrix [21]. The adjacency was represented in a topological overlap matrix (TOM), and dissimilarity was represented in a corresponding dissimilarity matrix (1 − TOM). The topological overlap provides a measure of biological gene similarity based on the association between pairwise gene co-expression. To classify genes with similar expression characteristics in gene co-expression modules, a hierarchical clustering dendrogram of the 1 − TOM matrix was built.

### Identification of clinically significant modules

The difference between the module-specific eigengenes (MEs) was calculated. A cutoff was selected for module dendrograms, and some modules were merged for further analysis [10]. Furthermore, the correlation between MEs and clinical trait information was evaluated to identify key modules that are significantly associated with PCC [21]. Next, the correlations of individual genes with clinical results were quantified by calculating the gene significance (GS) value [21]. Module significance (MS) was defined as the average GS for all genes in a module. In general, modules with the MS ranking of first or second were considered to be candidates for association with clinical characteristics. Overlapping genes between the DEGs and module genes were extracted from the co-expression network, and the genes closely related to the clinical phenotype of PCC were used to classify possible prognostic genes in a Venn diagram by the R-package [22].

### Gene ontology (GO) and pathway enrichment analysis for genes of interest

To gain deeper insight into the role of the overlapping genes identified as described above, GO enrichment analysis was performed with classification according to biological process, cellular component, and molecular function designations; and Kyoto Encyclopedia of Genes and Genomes (KEGG) pathway analysis was carried out using an R package cluster profile [23]. Functional categories and pathways were enriched using a cutoff of *p* < 0.05, and the top 10 GO categories were selected.

### Construction of a PPI network and screening of hub genes

The online tool STRING (Search Tool for the Retrieval of Interacting Genes), designed to predict functional interactions between proteins, has been used to create PPI networks for selected genes [24]. Genes with a score of ≥0.4 were selected using the STRING database to create a Cytoscape (v3.7.2) for visualization of the network model [25]. Maximal Clique Centrality (MCC) has been identified as a powerful index for detecting center nodes inside a network of co-expression [26]. Therefore, CytoHubba, a plugin at Cytoscape [26], was employed to measure each node’s MCC. The 10 genes with the highest MCC values in this analysis were identified as core genes.

### Survival analysis and prognostic values of hub genes

We calculated OS as an endpoint using the R packages survival and survminer. Survival curves were developed using the Kaplan-Meier method in R. In addition, the online platform GEPIA2 was used to calculate the correlation of DFS and hub genes expressed in patients with PCC [27]. Our survival analysis only included patients who had completed all follow-up examinations. The median expression values of hub genes were compared, and the samples were grouped into high-expression and low-expression groups. Survival-related hub genes with log-rank *p*-value significantly <0.05 were identified. Next, key survival genes and other prognostic ­predictors (age, gender, stage, and grade) were analyzed by univariate and multivariate COX analysis to assess the robustness of these genes compared with other prognostic indicators and to determine whether the key hub genes could be used as independent prognostic factors for PCC.

### Validation of the HPA database for protein expressions in survival-related hub genes

The HPA database (https:/www.proteinatlas.org/) is a comprehensive resource that enables researchers to access a wide range of transcriptional and proteomic data from different tissues and cells [28]. Moreover, protein expression patterns based on immunohistochemistry (IHC) have become a universal immunostaining application for the determination of the relative protein position and abundance [29]. Therefore, HPA was used to determine the abundance of proteins encoded by survival genes in PCC and control tissues.

### Ethics statement

This study protocol was reviewed and approved by the Hebei General Hospital Ethics Committee (No:202041). All patient data included in this study were de-identified.

## RESULTS

### Construction of PCC co-expression modules

**FIGURE 2:**
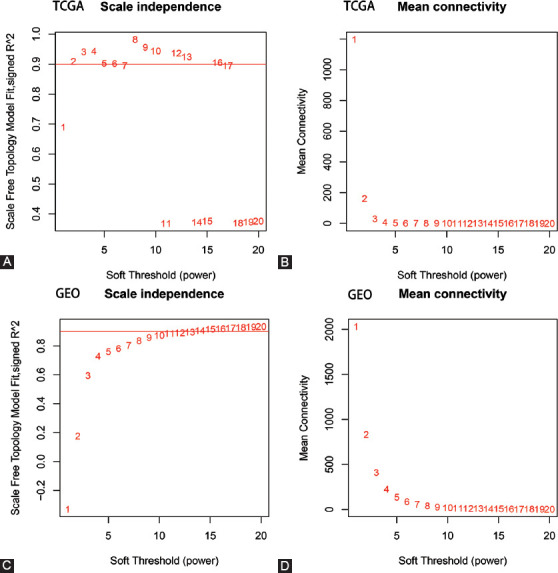
Identification of soft threshold weights by WGCNA. (A-D) Scale-free fitting index and average connectivity analysis of different soft-threshold weights of data from TCGA-PAAD (panels A and B) and GSE16515 (panels C and D).

**FIGURE 3:**
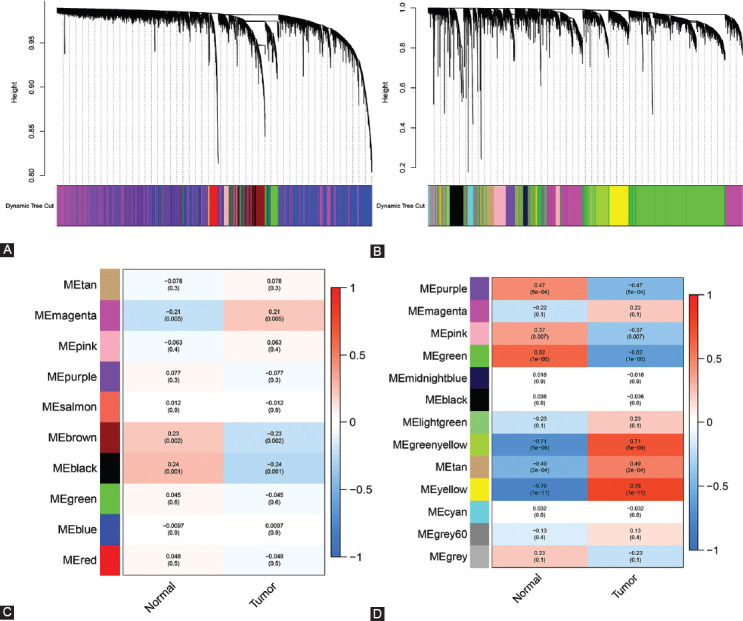
Relationships between PCC co-expression modules from TCGA-PAAD and GEO-PCC datasets. (A) Hierarchical gene clustering of TCGA co-expression modules according to the 1 − TOM matrix. Each module is color-coded. (B) Module-trait diagram for TCGA co-expression modules. Columns are colors and rows represent clinical features. (C) Hierarchical gene clustering of GEO co-expression modules according to the 1 − TOM matrix. Each module is color-coded. (D) Module-trait diagram for GEO co-expression modules. Columns are colors and rows represent clinical features.

To identify sets of genes that are co-expressed in PCC, we used WGCNA to sort genes from TCGA and GEO into modules. When the soft threshold values of β = 2 and 9 were selected, the connectivity between genes conformed to the distribution of the scale-free network (Figure 2A-D). We employed hierarchical clustering and dynamic branch cutting to recognize different co-expression modules of PCC and represented them by different colors. Ten modules of data from TCGA-PAAD (Figure 3A) and 12 modules from GSE16515 (Figure 3B) were detected after the fusion of related modules. Table S1 lists the number of genes present in the co-expression modules. To evaluate the correlation between each module and two clinical characteristics (cancer and normality), we plotted a heat map of module-trait relationships. The TCGA-PAAD magenta module and the GSE16515 yellow module represented the greatest association with the normal tissue (magenta module: *r* = −0.21, *p* = 0.005; yellow module: *r* = −0.78, *p* = 1e−11) (Figure 3C-D).

### Identification of genes between the DEG lists and co-expression modules

**FIGURE 4:**
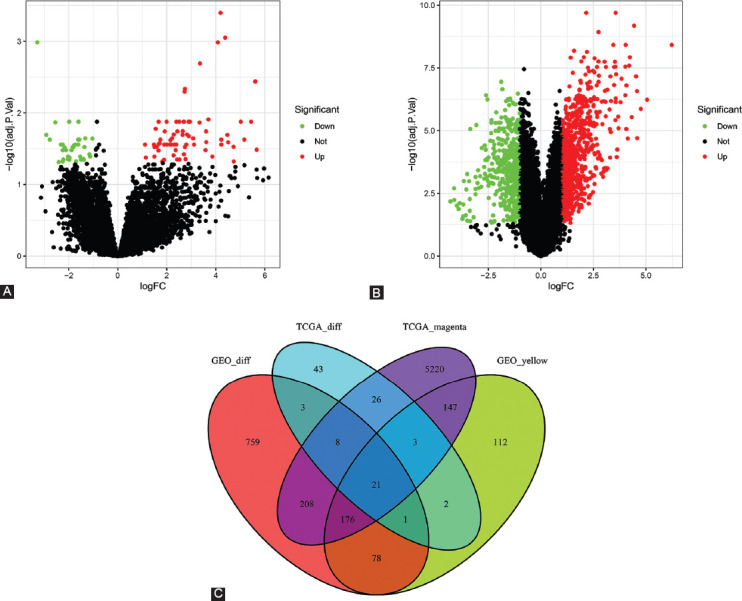
Detection of differentially expressed genes (DEGs) in PCC from the GSE16515 and TCGA datasets. (A-B) Volcano plots representing DEGs from the TCGA dataset (panel A) and the GSE16515 dataset (panel B). (C) The Venn diagram showing the intersection of genes between co-expression modules and DEG lists.

To further evaluate the differential expression pattern in PCC, we identified sets of DEGs. After data preprocessing and quality assessment through the limma package, 106 DEGs in TCGA-PAAD (Figure 4A) and 1253 DEGs in GSE16515 (Figure 4B) were identified to be dysregulated in tumor tissues. We further analyzed these DEGs according to their distribution in the co-expression modules. As shown in Figure 4C, the TCGA-PAAD magenta module had 5809 DEGs, and the GSE16515 yellow module had 540 DEGs. A total of 21 overlapping genes were identified as candidates for validation (Figure 4C).

### Functional enrichment analyses for 21 overlapping genes

**FIGURE 5:**
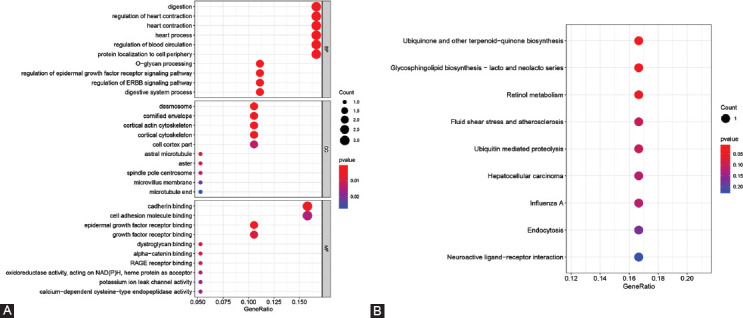
Functional annotation of pathways that are differentially activated in pancreatic cancer (PCC). (A) GO analysis of pathways modulated in PCC. (B) KEGG pathways enriched in PCC. The color represents the modified *p*-values. The scale of the spots indicates the number of genes involved.

To provide additional insight into the functional roles of the 21 dysregulated genes that overlapped in the DEG lists and co-expression modules, we performed KEGG and GO enrichment analyses using the clusterProfiler package. Several GO-enriched gene sets were observed (Figure 5A). Genes in the biological process category were primarily concentrated in O−glycan processing, regulation of the epidermal growth factor receptor signaling pathway, digestive system process, regulation of the ERBB signaling pathway, and protein localization to the cell periphery. In the cellular component category, enriched components included desmosomes, cell cortex part, cornified envelope, cortical cytoskeleton, and microvillus membrane. Moreover, in the molecular function category, cadherin binding, cell adhesion molecule binding, and epidermal growth factor receptor binding were the top functions of the 21 genes. In KEGG enrichment analysis, the genes were enriched in the biosynthesis of ubiquinone and other terpenoid-quinone, glycosphingolipid biosynthesis – lacto and neolacto series, and retinol metabolism (Figure 5B).

### PPI network construction and hub gene identification

**FIGURE 6:**
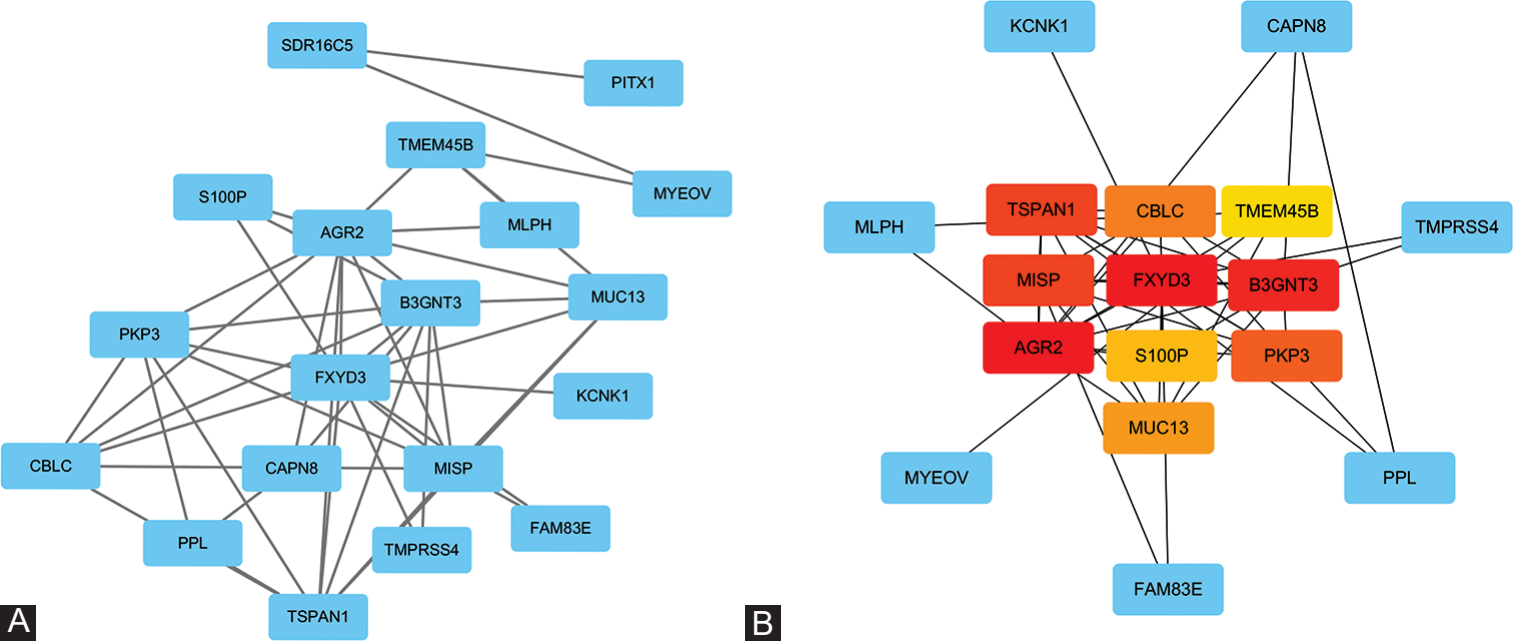
Construction of a PPI network and selection of the candidate core genes. (A) PPI network of intersecting genes from the Venn diagram. The genes are represented by the blue nodes. Edges indicate associations of interactions among nodes. (B) PPI network hub gene recognition using MCC. Genes with the highest MCC scores are red nodes; genes with the lowest MCC scores are yellow nodes.

For a more comprehensive understanding of the functions of the 21 overlapping genes, we constructed a PPI network. The network contained 19 nodes and 46 edges (Figure 6A). Further analysis identified core genes within the PPI ­network (Figure 6B). The 10 genes with the highest MCC scores, including Tetraspanin-1 (TSPAN1), E3 ubiquitin-protein ligase CBL-C (CBLC), transmembrane protein 45B (TMEM45B), mitotic interactor and substrate of PLK1 (MISP), FXYD domain-containing ion transport regulator 3 (FXYD3), beta-1,3-N-acetylglucosaminyltransferase-3 (B3GNT3), anterior gradient protein 2 (AGR2), Plakophilin-3 (PKP3), S100P, and mucin-13 (MUC13), were identified as Hub genes.

### Survival analysis and prognostic values of hub genes

**FIGURE 7:**
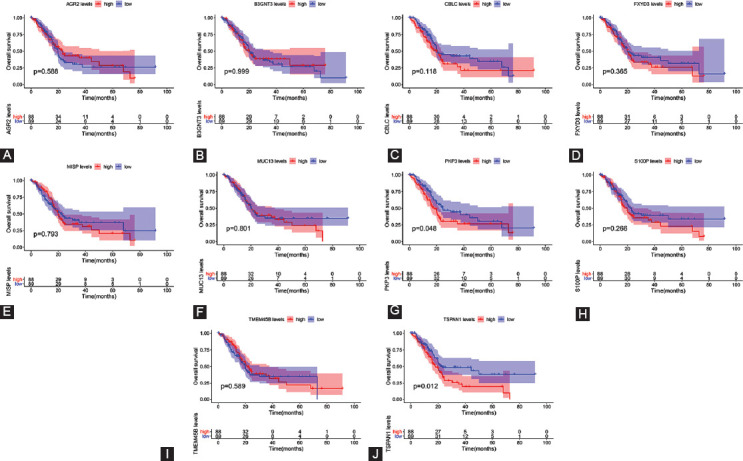
Correlation between the expression of the top 10 core genes and OS in PCC patients based on the GEPIA2 database. (A-J) ARG2, B3GNT3, CBLC, FXYD3, MISP, MUC13, PKP3, S100P, TMEM45B, and TSPAN1 in PCC survival analysis. *p* < 0.05 was defined as a statistically significant difference.

**FIGURE S1:**
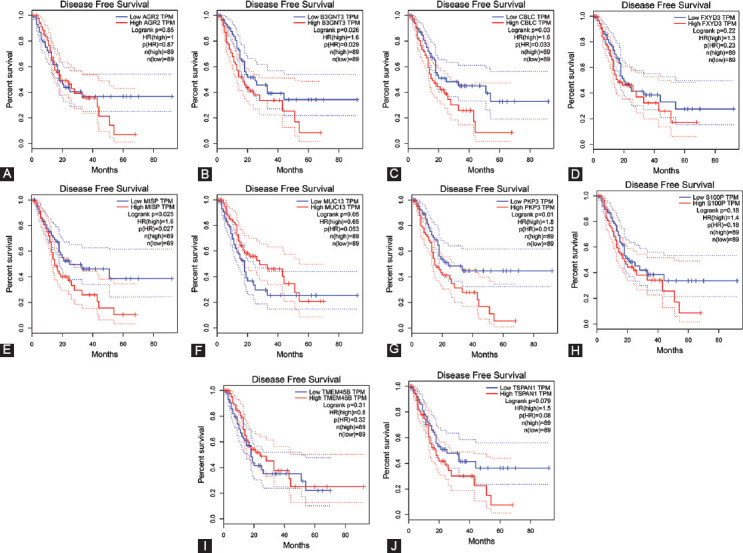
Correlation between the expression of the 10 core genes and DFS in PCC patients based on the GEPIA2 database. (A-J) Survival analysis of ARG2, B3GNT3, CBLC, FXYD3, MISP, MUC13, PKP3, S100P, TMEM45B, and TSPAN1 in PCC. Log-rank *p* < 0.05 was defined as a statistically significant difference.

**FIGURE S2:**
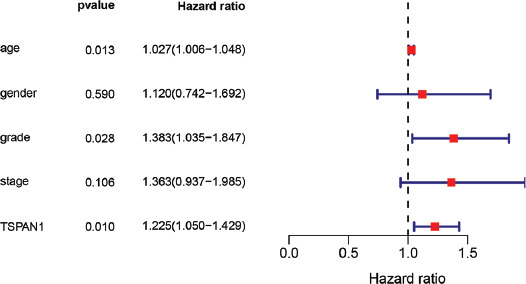
Univariate Cox regression analysis of TSPAN1 with other factors (age, gender, grade, and stage) in PCC patients.

To evaluate the clinical value of Hub gene expression, we determined whether they may be associated with survival or prognosis of PCC patients (Figure 7). Notably, high expression levels of TSPAN1 were significantly linked to the poor OS in PCC (*p* < 0.05) (Figure 7J). Although no substantial difference was found in the TSPAN1 expression level for DFS in PCC patients (*p* > 0.05) (Figure S1J), univariate and multivariate COX analysis outcomes indicated that TSPAN1 could serve as an independent prognostic factor for PCC patients (Table S2, Figure S2 and 8). These results suggest that TSPAN1 may represent a novel biomarker for PCC.

**FIGURE 8:**
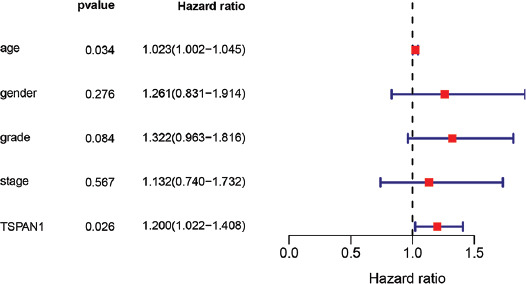
Multivariate COX regression analysis of TSPAN1 with other factors (age, gender, grade, and stage).

### Validation of the HPA database for core survival genes

**FIGURE 9:**
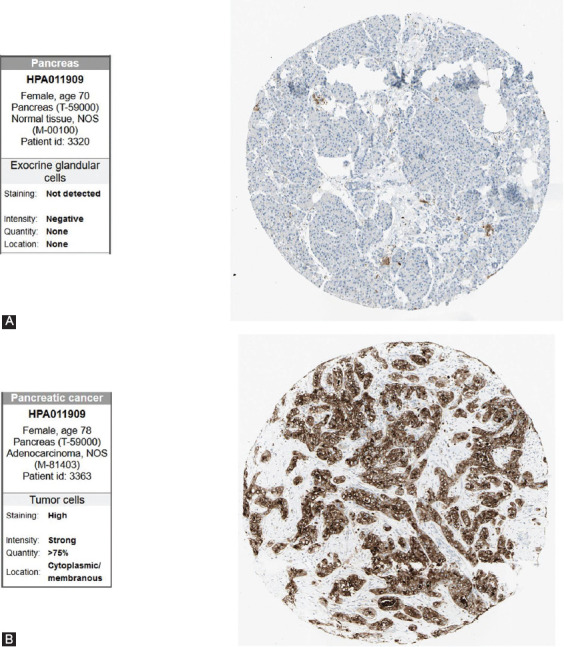
IHC of TSPAN1 in tumor tissues from the HPA database. (A) Protein levels of TSPAN1 in normal exocrine glandular cells tissues (antibody HPA011909; staining: Not detected; intensity: Negative; quantity: None). (B) Levels of TSPAN1 protein in PCC tissues (antibody HPA011909; staining: High; intensity: Strong; quantity: >75%).

To verify the enhanced expression of TSPAN1 in PCC, we accessed IHC data from the HPA database. The TSPAN1 protein levels were considerably higher in tumor tissues relative to healthy tissues (Figure 9). Therefore, these findings confirm that elevated TSPAN1 expression, as determined at both the mRNA and protein levels, is aligned with worse prognosis and lower OS in PCC patients.

## DISCUSSION

PCC, a common digestive tumor with a high degree of malignancy, tends to be aggressive and easily exacerbated by local nervous and vascular invasion. PCC tumors can form distal metastases in the early stages of cancer and often become resistant to traditional treatments such as chemotherapy and radiotherapy, making PCC prognosis extremely poor (5-year OS rate of <7%) [30]. Although many breakthroughs have been made for diverse cancers, the development of a PCC drug remains challenging [31]. With the advent and advancement of targeted therapies and precise medication, traditional histopathological evaluation and diagnosis are becoming increasingly outdated. The most common type of mutation in PCC patients is the KRAS mutation. The KRAS gene mutation plays a major role in the occurrence and development of PCC, with a rate as high as 90% [7]. KRAS pathway-targeted therapies for PCC have been explored, but current limitations involving drug resistance and safety considerations hinder their applicability. Therefore, to promote the development of precision medicine, including individualization and standardization of targeted drugs, we need to continue to explore new clinical survival targets for diagnosis, prediction, and treatment of PCC patients. Research involving genomics and transcriptomics has the potential to provide reliable and detailed information for clinical precision therapy, to extend patient survival, and to act as a guide for new drug development, including target selection for therapeutic trials and population screening. Matching different molecular subtypes to clinical drugs and treatment regimens have the potential to advance PCC therapy. Therefore, we used advanced bioinformatics methods and transcriptional and clinical data from curated databases to identify new potential molecular targets.

In this study, we identified 21 genes with consistent expression patterns using integrated WGCNA and DEG analysis. As indicated by GO functional enrichment results, the function of these 21 genes includes regulation of epidermal growth factor receptor (EGFR) signaling, cadherin binding, and cell adhesion molecule binding. Notably, EGFR (also known as ERBB1 and HER1) is overexpressed in 90% of PCC cells [32]. EGFR is a transmembrane receptor tyrosine kinase [33] that belongs to the ERBB family of cell surface receptor tyrosine kinases [34]. Binding of EGF to EGFR induces binding to other ERBB homologous or heterologous dimers. EGFR then induces receptor phosphorylation and activation of downstream effect molecules, including, for example, the RAS−RAF−MEK−ERK MAPK and PI3K−AKT mTOR pathways, and finally leading to cell proliferation. Furthermore, classical cadherin, which is a cell surface glycoprotein, mediates calcium-dependent cell adhesion in a homotypic manner [35,36]. The adhesion regulation function of cadherins requires interaction between beta-catenin and the actin cytoskeleton. During invasion and metastasis of tumor cells, the conversion of the cadherin isoform from E-type cadherin to N-type cadherin is related to epithelial-to-mesenchymal transition [37,38]. In particular, changes in the expression of E-cadherin in the pancreas contribute to the development of human intraepithelial pancreatic neoplasia [39].

Our KEGG enrichment data further suggest that the 21 overlapping genes perform biological roles in ubiquinone and another terpenoid-quinone biosynthesis, glycosphingolipid biosynthesis-lacto, and neolacto series, and retinol metabolism. Ubiquinone, also known as coenzyme Q, plays a central role in the mitochondrial electron transport chain, is involved in the production of mitochondrial oxidative phosphorylation and reactive oxygen species, and acts as a pivotal mediator of the pathogenesis of tumors [40]. Relevant studies have shown that ubiquinone can exert anti-tumor activity by promoting tumor cell proliferation and apoptosis [41], migration and invasion [42], and aerobic glycolysis [40]. Furthermore, work by Gehrmann et al. has demonstrated that the glycosphingolipid Gb3 facilitates tumor-specific Hsp70 plasma membrane localization [43]. Levels of HSP70 (a major stress-inducing member of the HSP70 family) on the plasma membrane have been considered as a prognostic indicator of OS in leukemia, lower rectal, and non-small cell lung carcinomas; however, it is unclear why tumors, but not healthy cells, present HSP70 on their cell surface, and the effect of the HSP70 membrane on cancer incidence remains to be clarified. Nevertheless, these results support a potential role for Gb3 in PCC prognosis.

We further identified 10 core genes (AGR2, FXYD3, B3GNT3, MISP, TSPAN1, PKP3, CBLC, MUC13, S100P, and TMEM45B) that were up-regulated in PCC tissues relative to healthy controls based on MCC evaluation results. Among them, increased expression of TSPAN1 was significantly associated with the poor OS rate of PCC. According to our COX analysis results, TSPAN1 also serves as an independent prognostic factor. TSPAN1 is a membrane glycoprotein and a member of a superfamily of transmembrane proteins (TM4SF) that have 4 members. While TM4SF has been studied only recently, its function in tumor invasion and metastasis has begun to be recognized. TSPAN1 has been shown to cause cancer cell proliferation and angiogenesis by switching cell division signals and inducing differentiation or dedifferentiation of cells [44]. According to prior research, TSPAN1 is widely expressed in gastric, lung, liver, and esophageal cancers [45-47]. As discussed above, beta-catenin is likely to have biological functions in the growth of PCC, and consistently, silencing of TSPAN1 has been shown to facilitate Smad2/3 phosphorylation and stabilize beta-catenin [48]. In addition, Hou et al. demonstrated that positive immunostaining of TSPAN1 is substantially associated with metastasis of the lymph node, TNM stage, and poor prognosis in PCC [49]. Of note, retinol metabolism and dissemination also play significant roles in the formation and evolution of tumors [50]. Our findings raise the possibility that TSPAN1 can interfere with the pathogenesis of PCC through the retinol metabolism pathway. Tian et al. demonstrated that the production of TSPAN1 in tumor tissues of PCC is dramatically higher than that of healthy tissues and that silencing of TSPAN1 reduces cell migration and invasion [51]. Furthermore, Wang et al. validated the oncogenic role of TSPAN1 in PCC, showing that TSPAN1 contributes to cell proliferation, migration, invasion, and tumorigenesis [52]. Zhang et al. also demonstrated that TSPAN1 up-regulates MMP2 through PLCg to promote PCC cell migration and invasion [53]. Therefore, our results demonstrating that TSPAN1 is overexpressed in tumor tissues but not healthy tissues reveal a clear link with survival in PCC patients that are compatible with previous findings.

## CONCLUSIONS

In summary, we identified gene co-expression modules and Hub genes linked to the progression and poor prognosis of PCC to guide further research, with potential benefits in the development of novel therapeutics. The present study does, however, have some limitations that should be acknowledged. First, although we did perform a thorough bioinformatics review to classify potential genes for diagnosis of PCC, the data may not be reliable for patients of every PCC subtype. Second, our research was constrained by the availability of experimental data. Confirmation using large-scale studies with subtype analysis may help to bring further insight into the role of TSPAN1 and other genes in PCC.

## Figures and Tables

**SUPPLEMENTARY TABLE 1:**
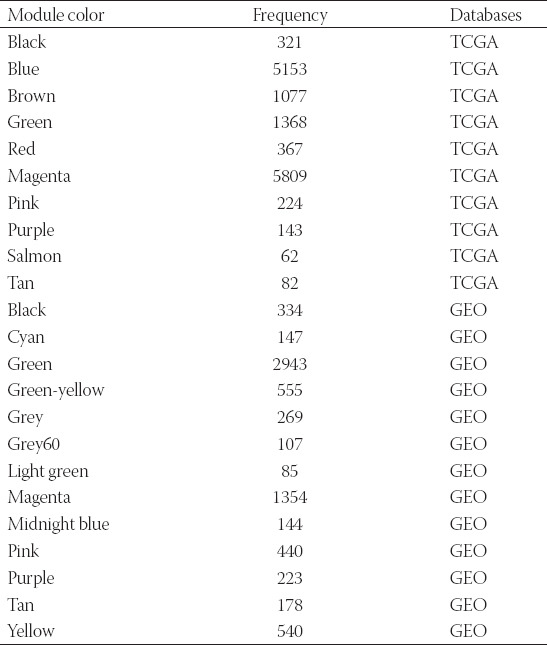
The number of genes in the co-expression modules from various databases.

**SUPPLEMENTARY TABLE 2:**
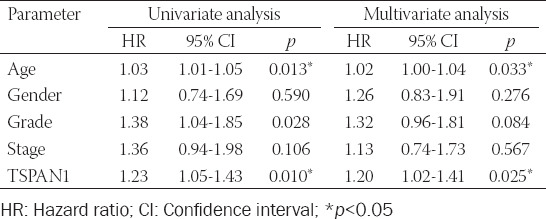
Univariate and multivariate COX analyses of TSPAN1 expression with other factors in PCC patients.
